# Relationship between Functional and Structural Changes in Diabetic Vessels in Optical Coherence Tomography Angiography

**DOI:** 10.1038/srep29064

**Published:** 2016-06-28

**Authors:** Yuko Miwa, Tomoaki Murakami, Kiyoshi Suzuma, Akihito Uji, Shin Yoshitake, Masahiro Fujimoto, Tatsuya Yoshitake, Yukino Tamura, Nagahisa Yoshimura

**Affiliations:** 1 Department of Ophthalmology and Visual Sciences, Kyoto University Graduate School of Medicine, Kyoto, Japan

## Abstract

The decorrelation signals in optical coherence tomography angiography (OCTA) are derived from the flow of erythrocytes and concomitantly delineate the retinal vasculature. We compared the structural and functional characteristics of vascular lesions visualized in fluorescein angiography (FA), OCTA, and en-face OCT images in 53 eyes (28 patients) with diabetic retinopathy (DR). The foveal avascular zone (FAZ) areas in OCTA images in the superficial layer almost corresponded to those in FA images. The FAZ areas in the en-face OCT images in the superficial layer were smaller than those in the FA images and correlated with each other, which agreed with the finding that en-face OCT images often delineated the vascular structure in the nonperfused areas in FA images. Microaneurysms appeared as fusiform, saccular, or coiled capillaries in OCTA images and ringed, round, or oval hyperreflective lesions in en-face OCT images. OCTA and en-face OCT images detected 41.0 ± 16.1% and 40.1 ± 18.6%, respectively, of microaneurysms in FA images, although both depicted only 13.9 ± 16.4%. The number of microaneurysms in FA images was correlated with that in OCTA and en-face OCT images. Comparisons of these modalities showed the associations and dissociations between blood flow and vascular structures, which improves the understanding of the pathogenesis of DR.

Diabetic retinopathy (DR), a diabetic microangiopathy, often leads to severe visual loss in working-age patients^1^,^2^. Capillary nonperfusion especially impairs the nutrition of the neuroglial tissues in the retinal parenchyma, and the resultant hypoxia increases expression of vascular endothelial growth factor (VEGF), which promotes both angiogenic responses and vascular permeability^3^,^4^, and causes ischemic maculopathy, proliferative diabetic retinopathy (PDR), and diabetic macular edema (DME). Fluorescein angiography (FA), the gold standard for evaluating the retinal vasculature, shows both morphologic and functional changes in the blood vessels in DR. Fluorescein dye in the plasma delineates vascular lesions including loss of the capillary beds, and the dye leakage suggests dysfunction of the tightly regulated blood-retinal barrier^5^,^6^,^7^.

Retinal sectional images using spectral-domain optical coherence tomography (SD-OCT) show large retinal vessels, i.e., vascular walls with higher OCT reflectivity and an hourglass shape within the lumen depending on the flow of erythrocytes^8^,^9^. OCT shows the qualitative and quantitative changes in the retinal parenchyma of the nonperfused areas, suggesting neuroglial structural changes and indirect proof of nutritional deficiency^10^,^11^,^12^. Clinical application of OCT angiography (OCTA) has improved the understanding of three-dimensional vascular lesions in several chorioretinal vascular diseases^13^,^14^,^15^,^16^. Decorrelation signals in OCTA images may depend on the movement of blood cells and resultantly delineate the morphologic and functional changes in the retinal vessels accompanied by blood flow. Recent publications have documented that OCTA clearly depicts the diabetic vascular changes, i.e., microaneurysms, venous beading, intraretinal microvascular abnormalities, neovascularization, and nonperfused areas in DR^13^,^17^,^18^. Despite the clinical relevance of OCTA imaging, several lines of artifacts and vasculature without the movement of blood cells must be considered^19^.

In the current study, we quantified the foveal avascular zone (FAZ) areas and the number of microaneurysms in FA, OCTA, and en-face OCT images, and evaluated their relationship among the vascular changes in the images obtained using these modalities in DR.

## Results

### Comparison between OCTA and En-face OCT

**Figure 1:**
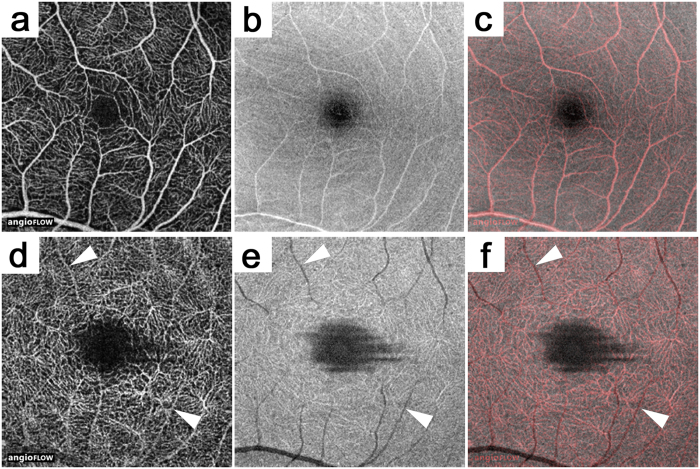
Comparison between OCTA and en-face OCT images in a healthy eye. The superficial (**a–c**) and the deep layers (**d–f**). (**a,d**) OCTA images. (**b,e**) En-face OCT images. (**c,f**) Merged images of OCTA (red) and en-face OCT (gray). The arrowheads indicate projection artifacts on OCTA images (**d**) and their corresponding vessel shadows (**e**).

Decorrelation signals depict objects moving in the retina, which enabled us to evaluate the retinal vasculature three-dimensionally. The vascular density in the deep layer was much higher than that in the superficial layer in the healthy eyes (Fig. 1a,b). Arterioles and venules especially often were accompanied by underlying shadows in the deep layers in the en-face OCT images, although the OCTA images showed higher decorrelation signals, referred to as projection artifacts (Fig. 1d,e)^19^. The artifacts in the OCTA images depend on the time-sequential differences of the OCT reflectivity beneath the vasculature, which suggested the clinical significance of the comparative studies between the OCTA and en-face OCT images for interpreting the three-dimensional vascular structures in individual eyes.

### Vascular Structures in En-face OCT Images in Nonperfused Areas in FA Images

**Figure 2:**
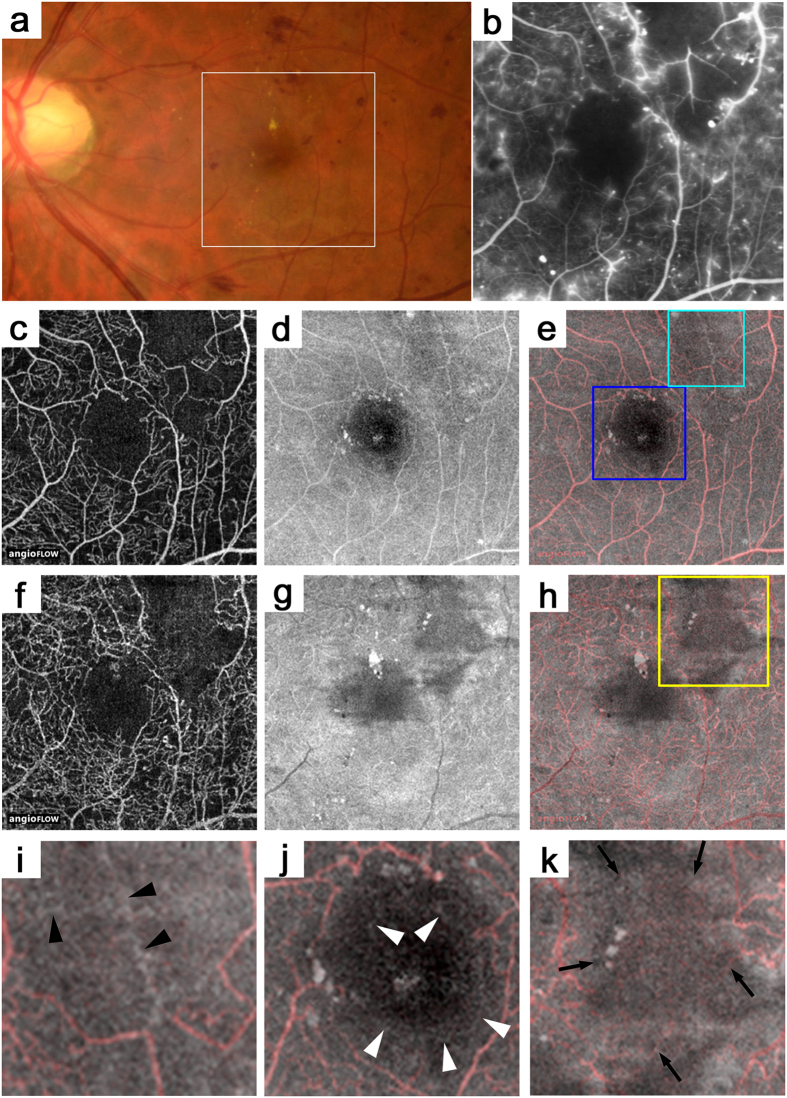
OCTA and en-face OCT images in the nonperfused areas in a 43-year-old patient with moderate NPDR. (**b**) The FA image that corresponds to the white square in the fundus photograph (**a**). OCTA (**c**,**f**), en-face OCT (**d,g**), or merged images of OCTA (red) and en-face OCT (gray) (**e,h**). The nonperfused areas in the superotemporal subfield in the FA images (**b**) almost correspond to those in the superficial layer in the OCTA images (**c**), whereas a cord-like structure is seen in the en-face OCT images (**d**,**e,i**). (**j**) The en-face OCT images also visualize capillaries (arrowheads) within the foveal avascular zone in the FA and OCTA images. (**i,j**) are magnified images of the light blue or deep blue square in (**e**). (**k**) The magnified image of the yellow square in (**h**). In the deep layer corresponding to the nonperfused areas in the FA image, dotted or dashed lines with faint decorrelation signals are seen in the OCTA images (arrows), whereas the en-face OCT image does not show the capillaries.

**Figure 3:**
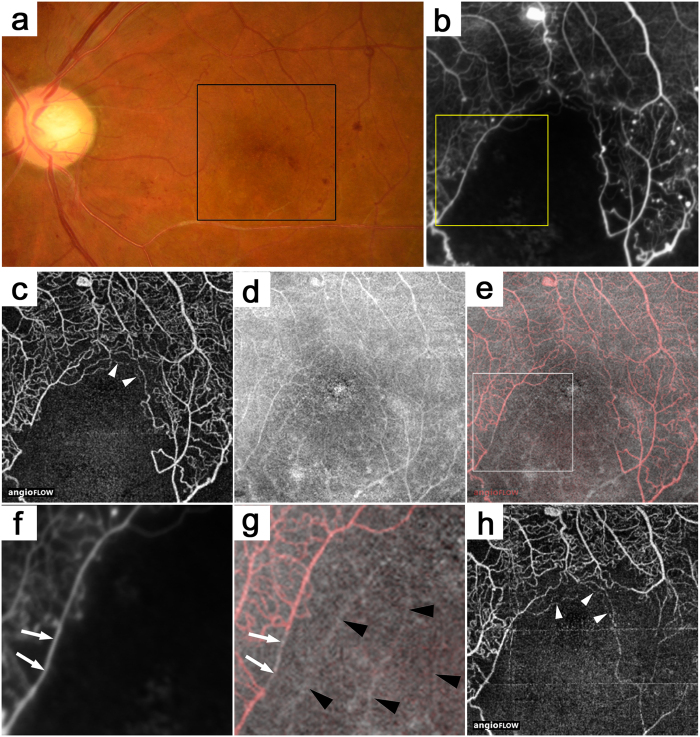
The inconsistency between the capillaries in the OCTA and en-face OCT images in the nonperfused areas in a 44-year-old patient with PDR. (**b**) A FA image that corresponds to the black square on the fundus photography (**a**). The superficial layer in the OCTA (**c**), en-face OCT (**d**), and merged (**e**) images (red = OCTA and gray = en-face OCT). (**f,g**) The magnified images of the yellow square in b and the white square in e, respectively. Cord-like structures (black arrowheads) in the en-face OCT image in the nonperfused areas in the FA or OCTA images. It is rare that capillaries in the FA images have no decorrelation signals in the OCTA images (arrows). The OCTA images before (**h**) and after (**c**) panretinal photocoagulation shows partial reperfusion in the inferotemporal subfield. The white arrowheads in c and h show dotted or dashed lines around the nonperfused areas.

The retinal capillaries usually appeared as continuous and curved lines in the OCTA images, whether the decorrelation signals were definite or faint, although dashed or dotted lines often were seen around the nonperfused areas (Figs 2f and 3c,h). These findings suggested various levels or patterns of blood cell movement compared to the continuous and homogeneous hyperfluorescence in the capillaries in the FA images. Diabetic eyes had capillaries in the FA images that almost corresponded to those in the superficial layer in the OCTA images, although FA alone rarely detected the blood vessels (Fig. 3f,g)^20^. In contrast, cord-like structures with higher reflectivity often were seen in the en-face OCT images in the nonperfused areas determined by OCTA or FA images (Figs 2j and 3g). This suggested a structural-functional inconsistency in the vasculature of diabetic retinas.

**Figure 4:**
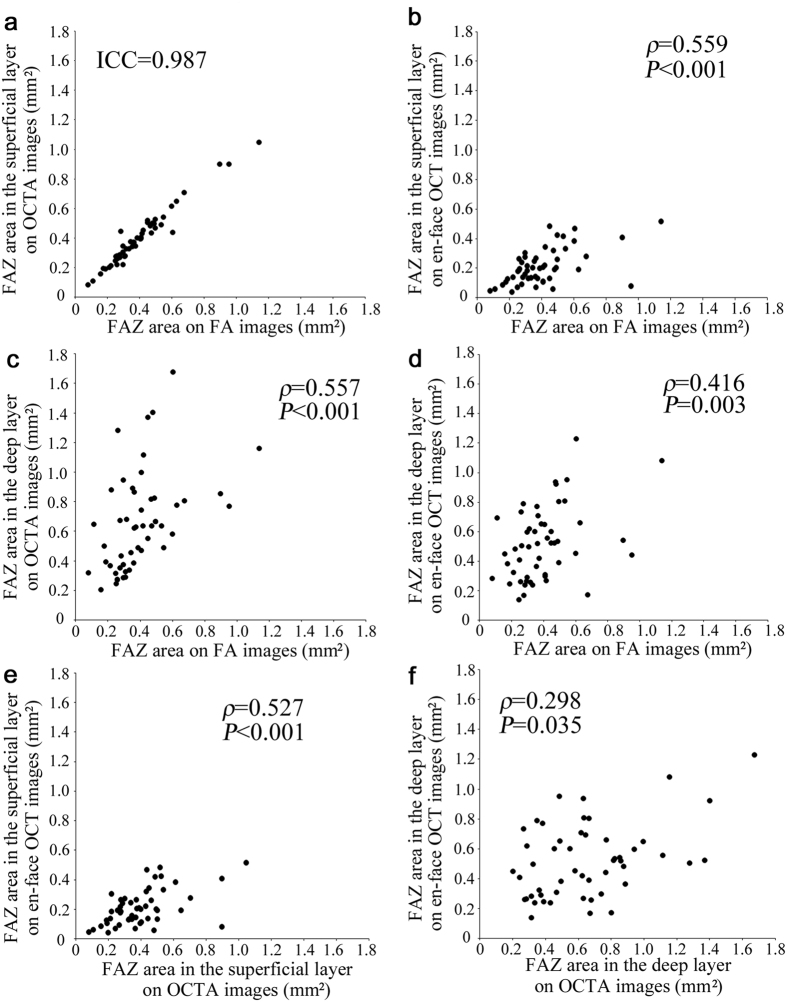
Association of the FAZ areas in the FA images with those in the OCTA and en-face OCT images in DR. Correlation of the FAZ areas on FA with those in the superficial layer on OCTA (**a**) and en-face OCT images (**b**) and those in the deep layer on OCTA (**c**) and en-face OCT images (**d**). (**e**,**f**) The relationship between the FAZ areas in the OCTA and en-face OCT images in the superficial or deep layer. ICC = intraclass coefficient.

We especially focused on the areas of the FAZs in the FA, OCTA, and en-face OCT images in 51 eyes, which had good intergrader compatibility (intraclass correlation coefficient [ICC], 0.970, 0.985, 0.944, 0.954, and 0.913 in the FAZ areas in the FA images, the superficial and deep layers in the OCTA images, and the superficial and deep layers in the en-face OCT images, respectively). The FAZ areas in the FA images were almost the same as those in the superficial layer in the OCTA images in eyes with DR (ICC = 0.987) (Fig. 4a). However, in the superficial slabs, the FAZs in the en-face OCT images were smaller than those in the OCTA and FA images (0.191 mm^2^ [interquartile range {IQR}, 0.126–0.268] vs. 0.371 mm^2^ [0.273–0.485] and 0.364 mm^2^ [0.277–0.476], *P* < 0.001 for both comparisons). There was a significant correlation between the FAZ sizes in the FA and OCT images in the superficial layers (*ρ* = 0.559, *P* < 0.001). In the deep layer, the innermost capillaries were not as definite in the OCTA and en-face OCT images, and the FAZ areas in the OCTA images were significantly larger (0.633 mm^2^ [0.378–0.820] vs. 0.504 mm^2^ [0.302–0.655], *P* = 0.003). The FAZ areas in the deep layer in the OCTA and en-face OCT images also were correlated modestly with those in the FA images (*ρ* = 0.557, *P* < 0.001 and *ρ* = 0.416, *P* = 0.003, respectively) (Fig. 4c,d). However, we had to carefully interpret these analyses, because the FAZ areas in the OCTA or en-face OCT images could be modulated by the segmentation errors at the boundary between the inner plexiform layer (IPL) and the inner nuclear layer (INL) (151/477 points [31.7%]) rather than those at the inner retinal surface (12/477 points [2.5%]).

### Microaneurysms in OCTA and En-face OCT Images

**Figure 5:**
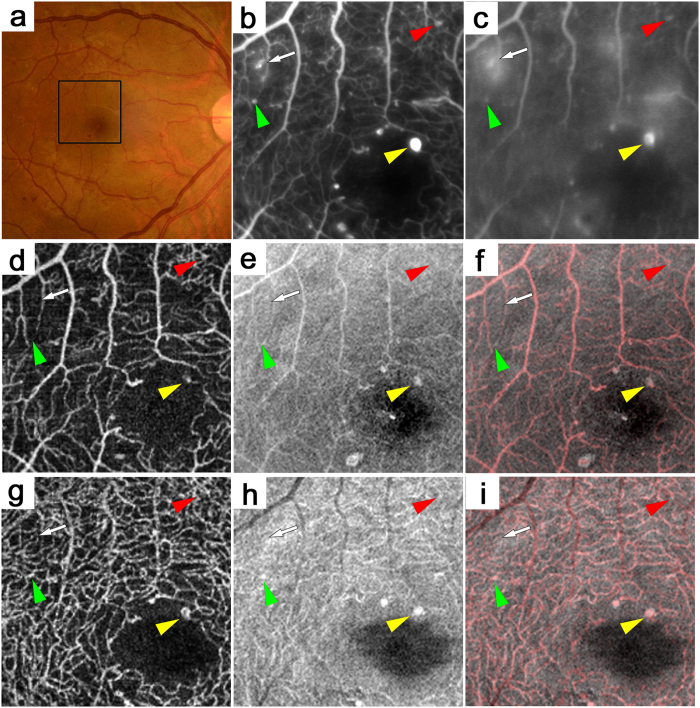
Microaneurysms in the OCTA and en-face OCT images in a 44-year-old patient with PDR. (**b,c**) The early- and late-phase FA images of the areas corresponding to the black square in the color fundus photograph (**a**) show microaneurysms as hyperfluorescent dots (arrow and arrowheads). Some microaneurysms are seen in the OCTA images alone (red arrowhead) or in the en-face OCT images alone (green arrowhead) or in both (yellow arrowhead). Others are not seen in either the OCTA or en-face OCT image (arrow). (**d**,**g**) OCTA images. (**e**,**h**) En-face OCT images. (**f**,**i**) Merged images of OCTA (red) and en-face OCT (gray). Superficial (**d–f**) or deep (**g–i**) layer.

**Figure 6:**
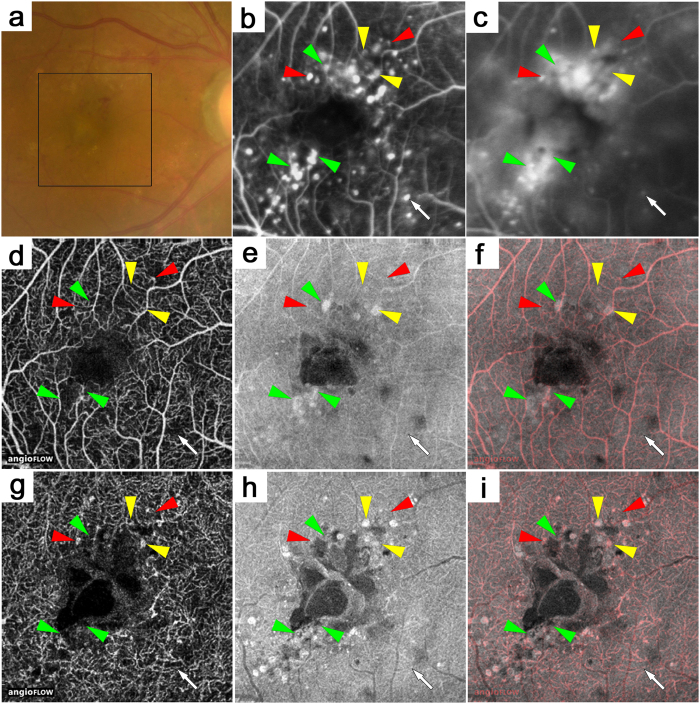
Microaneurysms in the superficial or deep layer adjacent to cystoid spaces in a 41-year-old patient with PDR and DME. The early- or late-phase (**b**,**c**) FA images of the areas corresponding to the black square in the color fundus photograph (**a**). Some microaneurysms are seen in the OCTA images alone (red arrowheads) and have various morphologies, i.e., fusiform, saccular, and coiled. Some microaneurysms are well-defined in the en-face OCT images alone (green arrowheads) or in both (yellow arrowheads). Others are not depicted in either the OCTA or en-face OCT image (arrow). Microaneurysms in either the superficial (**d**–**f**) or deep (**g**–**i**) layer are sometimes adjacent to cystoid spaces. (**d**,**g**) OCTA images. (**e**,**h**) En-face OCT images. (**f**,**i**) Merged images of OCTA (red) and en-face OCT (gray).

**Figure 7:**
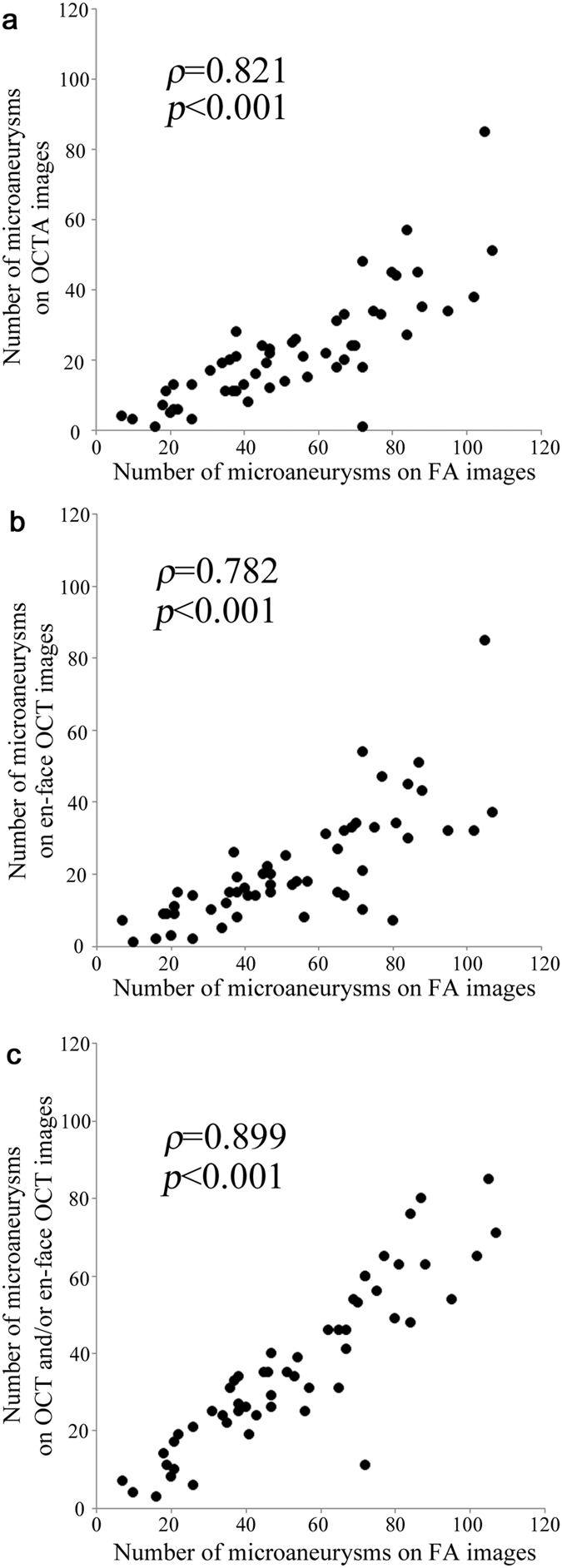
Association of microaneurysms in the FA with OCTA, en-face OCT, or OCTA and/or en-face OCT images in DR. Comparison of microaneurysms on fluorescein angiography (FA) with optical coherence tomography angiography (OCTA) (**a**), en-face optical coherence tomography (OCT) (**b**), or OCTA and/or en-face OCT images (**c**) in diabetic retinopathy.

We also investigated the microaneurysms in the OCTA and en-face OCT images that corresponded to the hyperfluorescent dots in the early and late phases. The decorrelation signals in the OCTA images showed a few morphologic patterns, i.e., fusiform, saccular, curved, and rarely coiled, compared to the homogeneous dot-like signals in the FA images (Figs 5d,g and 6d,g). Microaneurysms appeared in the en-face OCT images as highly reflective round, oval, or sometimes ringed lesions in DR, which agreed with recent studies about B-scan images (Figs 5e,h and 6e,h)^21^,^22^,^23^. We counted microaneurysms in the FA, OCTA, and en-face OCT images, which had good intergrader compatibility (ICC = 0.956, 0.939, and 0.917 in the FA, OCTA, and en-face OCT images, respectively), and compared microaneurysms in the OCTA or en-face OCT images to those in the FA images. Only 41.0 ± 16.1% of the microaneurysms in the FA images showed such lesions in the OCTA images in all 53 eyes. En-face OCT images delineated 40.1 ± 18.6% of the microaneurysms in the FA images. The numbers of microaneurysms in the OCTA and en-face OCT images were associated significantly (*ρ* = 0.821, *P* < 0.001and *ρ* = 0.782, *P* < 0.001, respectively) with the number in the FA images (Fig. 7a,b). Further studies showed that 13.9 ± 16.4% of microaneurysms were seen in both the OCTA and en-face OCT images. Either OCTA or en-face OCT images detected 67.3 ± 18.3% of the microaneurysms in the FA images, and the numbers of microaneurysms in either image were significantly correlated with that in the FA images (*ρ* = 0.899, *P* < 0.001) (Fig. 7c).

## Discussion

Capillary nonperfusion has a significant impact on visual impairment and leads to VEGF expression and concomitant pathogenesis in PDR and DME, although *in vivo* vascular pathophysiology in the nonperfused areas remains to be elucidated^3^,^4^,^10^,^11^,^24^,^25^,^26^,^27^,^28^. The three components of the retinal vasculature are plasma, blood cells, and vascular structures, which were clinically represented by the signals in the FA, OCTA, and OCT images, respectively. Since blood cells and plasma transport oxygen and nutrients through the vascular lumens, the current study showed the structural/functional association and dissociation in the retinal vasculature in the nonperfused areas and microaneurysms. Systematic evaluation using these modalities would improve the clinical interpretation of OCTA images in the retinal vasculature in diabetic patients.

The decorrelation signals in the OCTA images depend mainly on the movement of blood cells and show the retinal vasculature accompanied by the flow of erythrocytes^14^,^15^. Considering that the size of the blood cells was almost the same as the diameter of the capillaries, plasma moves between the blood cells in the capillaries^29^. This supports to some extent the coincidence of the capillary nonperfusion in the FA and OCTA images. The levels of the decorrelation signals varied, i.e., definite or faint, and depended on the speed or amounts of the blood flow, as discussed previously^19^. The capillaries sometimes appeared as dotted or dashed lines in the OCTA images. Erythrocytes with less plasticity, activated platelets, clotting systems, or increased interaction between the vascular cells and leukocytes as the result of diabetes may lead to transient plugging and concomitant segmental loss of decorrelation signals^25^,^26^,^27^. Compared to the various appearances of the decorrelation signals in the OCTA images, the FA images showed capillaries as homogeneous and continuous lines. We speculated that hyperfluorescent lines without decorrelation signals in the OCTA images suggested plasma flow with no blood cells or fewer motile blood cells (Fig. 3f,g).

In healthy eyes, en-face OCT images detected larger vessels and capillaries in the INL, although it remains ill-defined whether higher OCT reflectivity in vessels depends on vascular walls or blood cells^8^. Diabetic capillaries were detected more clearly in the OCT images, and the FAZ areas could be easily identified in the en-face OCT images in DR compared to the ill-defined parafoveal capillaries in healthy eyes. We speculated that remodeling in the capillaries increases their diameter, which allows better observation of the capillaries in the en-face OCT images. Histologic studies have documented thickened basement membranes in diabetic capillaries, which might increase the OCT reflectivity in diabetic capillaries^30^. However, the comparative study showed that the capillary density in the en-face OCT images was smaller than in the OCTA images. A few possible explanations are the lower detection ability of the en-face OCT images or projection artifacts from the superficial vessels in the OCTA images^19^. The OCT reflectivity of the retinal parenchyma in the superficial layer is relatively higher in the parafoveal areas, and the differences in the optical density decreased between the vasculature and neuroglial tissues. Therefore, the en-face OCT images could not clearly show the parafoveal capillaries in the superficial layer.

FA or OCTA images show the nonperfused areas in DR, although the images cannot determine if the capillary nonperfusion is transient or permanent. Interestingly, en-face OCT images visualized a cord-like structure that corresponded to the vascular structure in the nonperfused areas. This suggested reperfusion of the capillaries is possible after interventions. In fact, anti-VEGF therapy for DME improves the capillary perfusion in the macula^31^. We observed some eyes in which some portions of the nonperfused areas became reperfused after interventions (Fig. 3c,h). Pathophysiologic research has shown several mechanisms regarding transient nonperfusion in diabetic retinas, and histologic studies have documented acellular capillaries or hyalinized vessels as permanently obstructed^25^,^26^,^27^,^30^,^32^,^33^,^34^. Unfortunately, we could not determine whether the vascular structure in the nonperfused areas corresponded to transient or permanent obliteration. Further investigation using adaptive optics scanning laser ophthalmoscope combined with a split detector or an offset pinhole might elucidate whether or not these vascular structures have lumens and whether the capillary nonperfusion is transient or permanent^35^.

Ischemic maculopathy often has a great impact on the visual acuity in DR^10^,^24^. OCTA may have clinical feasibility for evaluating this pathogenesis, since the FAZs in the FA images corresponded to that in the superficial layer in the OCTA images^36^. Interestingly, en-face OCT images delineated the vascular structure in the FAZs in the FA and OCTA images in the superficial layer. This suggested that some areas of the FAZs may become reperfused through this vascular structure, which may explain the variability in the visual impairment in ischemic maculopathy. The FAZ areas in the en-face OCT images were smaller than and modestly correlated with those in the FA images, which may allow us to hypothesize that the vascular structure disappears after capillary nonperfusion rather than simultaneously with nonperfusion.

OCTA showed the various morphologies of the microaneurysms and detected only 41.0 ± 16.1% of microaneurysms in the FA images. Recent studies have reported that OCTA delineates microaneurysms less repeatably^19^. Some microaneurysms may have no erythrocytes or blood cells with less motility, which could not be visualized in the OCTA images, although the movement of erythrocytes is not constant in the microaneurysms^37^. Histologic studies have reported saccular or fusiform microaneurysms, and OCTA showed similar morphologies in some microaneurysms^30^,^34^,^38^,^39^. We also sometimes observed curved or coiled capillaries corresponding to hyperfluorescent dots in the FA images. The inconsistency between the microaneurysms in the FA and OCTA images may be explained by fluorescein dye filling without blood cells. Another possibility may be tissue staining of the vascular walls in the microaneurysms or surrounding tissues in the FA images, which could be independent of the movement of the erythrocytes. En-face OCT images showed some microaneurysms with better repeatability but detected only 40.1 ± 18.6% of the microaneurysms in the FA images. When the OCT reflectivity does not differ between the vascular walls and surrounding tissues, en-face OCT cannot clearly depict the microaneurysms. Further investigation is needed to clarify the characteristics of the leaky microaneurysms in the OCTA and en-face OCT images^6^.

We had to consider two major limitations, i.e., the generalizability and the segmentation error. In the current study, we investigated diabetic changes in a Japanese population; further studies should elucidate whether these findings are generalizable to other populations. Since the manufacturer’s software could not always detect the true boundary between the IPL and the INL, the incorrect segmentations of both the superficial and deep layers in the OCTA or en-face OCT images frequently occurred in eyes with intraretinal lesions. The FAZ areas in the superficial layer in the OCTA images were almost the same as those in the FA images, suggesting that the FAZ areas in the superficial layer might be correct to some extent. In contrast, the FAZ areas in the deep layer could be modulated by the segmentation errors in addition to the projection artifacts or flow void in cystoid spaces^19^.

In conclusion, we showed the association and dissociation between diabetic changes in OCTA and en-face OCT images that represented the flow of blood cells and vascular structures respectively. This supports their clinical feasibility and improves the understanding of the pathological changes in individual vascular components.

## Methods

### Patients

In this retrospective study, we reviewed 53 consecutive eyes of 28 patients with DR (mean age, 65 years [IQR, 47–72]; all patients with type 2 diabetes mellitus; 2 eyes with mild nonproliferative DR [NPDR], 16 with moderate NPDR, 16 with severe NPDR, and 19 with PDR), who visited the Department of Ophthalmology of Kyoto University Hospital from February 2015 to November 2015. Eyes with DR were included in the study if both FA and OCTA images of sufficient quality were acquired^5^. The exclusion criteria were the presence of other chorioretinal diseases; glaucoma or ocular hypertension; a history of any intervention for macular lesions within 6 months; a history of photocoagulation of the macular area; intraocular surgery other than cataract extraction; and cataract surgery within 6 months of study enrollment. Twenty-three eyes had center-involved DME, which was determined by two-dimensional mapping using Spectralis OCT (Heidelberg Engineering, Heidelberg, Germany) as shown previously^40^,^41^. All research and measurements adhered to the tenets of the Declaration of Helsinki. The ethics committee of our institution approved the study protocol. All participants provided written informed consent.

### Fluorescein angiography

The FA images of the macula were acquired using Heidelberg Retinal Angiography 2 (Heidelberg Engineering), followed by quantification of the areas of the FAZs, as described previously^42^. Briefly, the innermost capillaries around the fovea were identified, and the internal area was measured using ImageJ software (National Institutes of Health, Bethesda, MD). Two eyes with enlarged FAZs extending to the margin of the images (central 3 × 3 mm) were excluded from the measurements of the FAZ areas. We counted the numbers of microaneurysms on the FA images in the early- and/or late-phases within the areas corresponding to the central 3 × 3 mm areas obtained by Optovue RTVue XR Avanti (Optovue, Inc., Freemont, CA).

### Optical coherence tomography angiography

OCTA and OCT images of the central 3 × 3 mm were obtained simultaneously using the Optovue RTVue XR Avanti (Fig. 1). This instrument has a high A-scan rate of 70,000 scans/second, using a light source of approximately 840 nm and depicts motion-dependent angiography using the split-spectrum amplitude decorrelation angiography algorithm (SSADA), as described previously^14^. Briefly, two consecutive B-scans (M-B frames) were obtained at a fixed position before proceeding to the next. After processing the volume scans, the calculation of the decorrelation between the sequential images allowed detection of the motion of the blood cells and concomitantly constructed a motion contrast “angioflow” image.

We applied the segmentation processes to evaluate the en-face images of the superficial and deep capillary plexus layers using the manufacturer’s software^13^. The superficial slab images showed the superficial layer, which extended from the inner boundary 3 μm beneath the internal limiting membrane (ILM) to the outer boundary 15 μm beneath the IPL. The deep en-face images showed the deep layer from the inner boundary 15 μm beneath the IPL to the outer boundary 70 μm beneath the IPL. Histologic studies have reported that capillary plexuses reside mainly in the nerve fiber layer (NFL), ganglion cell layer (GCL), and the inner and outer borders of the INL^43^. Taken together, the superficial layer contains the capillary plexuses in the NFL and GCL, whereas the deep layer has capillaries in the inner and outer boundaries of the INL. Since the segmentation according to the manufacturer’s software is not as accurate as desired, we manually evaluated the error rates of the segmentation at the boundary between the vitreous and the ILM and that between the IPL and the INL. We selected vertical and horizontal sectional images dissecting the fovea, and compared the true boundaries to those determined by the software at nine points including the fovea, 0.5 and 1 mm from the fovea in the nasal, temporal, superior, and inferior subfields.

We measured the areas of the FAZ in a 3 × 3-mm square in the OCTA and en-face OCT images. The FAZ areas in the OCTA images were measured as previously described for the FA images, because the higher contrast in the OCTA images enabled the quantification^42^. We further evaluated the areas of the FAZ in the en-face OCT images, which were acquired simultaneously with the OCTA images. Recent studies have reported that the vascular walls are delineated as highly reflective structures in the SD-OCT images, which allowed us to define a cord-like structure with higher OCT reflectivity as a vascular structure in the en-face OCT images, whether or not it corresponded to the vasculature in the FA images^8^. We thus measured the FAZ areas circumscribed by the innermost vascular structure at the fovea in the en-face OCT images.

We also compared the microaneurysms in the OCTA and en-face OCT images to those in the FA images. Briefly, microaneurysms, seen as hyperfluorescent dots in FA images in the early and/or late phases, were the gold standard for further investigation. A recent study showed that OCTA depicted microaneurysms as demarcated saccular or fusiform shapes of focally dilated capillary vessels in either the superficial or deep layer^13^. We also found that the curved or coiled capillaries in the OCTA images sometimes corresponded to the microaneurysms in the FA images. However, previous reports have shown that microaneurysms appeared as ringed or oval lesions with higher reflectivity in the retinal sections of SD-OCT images^21^,^22^,^23^, indicating that these lesions corresponded to ringed, round, or oval structures with high OCT reflectivity in the thicker slab images. Diabetic retinas often have hyperreflective lesions including intraretinal hemorrhages, fibrin, and hyperreflective foci^23^,^44^. We thus carefully compared the hyperreflective lesions in the en-face OCT images to hyperfluorescent dots in the FA images and defined the microaneurysms in the en-face OCT images in this study. We counted the microaneurysms in a central 3 × 3-mm square on the FA, OCTA, and en-face OCT images.

### Statistical Analysis

Two masked graders evaluated the quantitative parameters, and the average was used for further analyses. The graders further assessed the qualitative findings, and in the event of a disagreement, a third grader participated. The results are expressed as the median (IQR). The statistical correlation was evaluated using Spearman’s correlation coefficient. To assess the agreements, we calculated the ICC. The differences between matched parameters were determined by Wilcoxon’s signed-rank test. *P* < 0.05 was considered significant.

## Additional Information

**How to cite this article**: Miwa, Y. *et al*. Relationship between Functional and Structural Changes in Diabetic Vessels in Optical Coherence Tomography Angiography. *Sci. Rep*. **6**, 29064; doi: 10.1038/srep29064 (2016).
